# miR-122-mediated translational repression of PEG10 and its suppression in human hepatocellular carcinoma

**DOI:** 10.1186/s12967-016-0956-z

**Published:** 2016-07-02

**Authors:** Yu-Chiau Shyu, Tung-Liang Lee, Mu-Jie Lu, Jim-Ray Chen, Rong-Nan Chien, Huang-Yang Chen, Ji-Fan Lin, Ann-Ping Tsou, Yu-Hsien Chen, Chia-Wen Hsieh, Ting-Shuo Huang

**Affiliations:** Community Medicine Research Center, Keelung Chang Gung Memorial Hospital, Keelung 204, Taiwan; Institute of Molecular Biology, Academia Sinica, Nankang, Taipei 115, Taiwan; Department of Experimental Radiation Oncology, University of Texas MD Anderson Cancer Center, Houston, TX USA; Department of Pathology, Keelung Chang Gung Memorial Hospital, Keelung 204, Taiwan; Department of Medicine, College of Medicine, Chang Gung University, Kwei-Shan, Taoyuan 259, Taiwan; Department of Gastroenterology and Hepatology, Keelung Chang Gung Memorial Hospital and University, Keelung 204, Taiwan; Department of General Surgery, Keelung Chang Gung Memorial Hospital, Keelung 204, Taiwan; Central Laboratory, Shin-Kong Wu Ho-Su Memorial Hospital, Taipei 111, Taiwan; Institute of Biotechnology in Medicine, National Yang Ming University, Taipei 112, Taiwan; Department of Chinese Medicine, College of Medicine, Chang Gung University, Kwei-Shan, Taoyuan 259, Taiwan

**Keywords:** miR122, PEG10, HCC

## Abstract

**Background:**

Hepatocellular carcinoma (HCC), a primary liver malignancy, is the most common cancer in males and fourth common cancer in females in Taiwan. HCC patients usually have a poor prognosis due to late diagnosis. It has been classified as a complex disease because of the heterogeneous phenotypic and genetic traits of the patients and a wide range of risk factors. Micro (mi)RNAs regulate oncogenes and tumor suppressor genes that are known to be dysregulated in HCC. Several studies have found an association between downregulation of miR-122, a liver-specific miRNA, and upregulation of paternally expressed gene 10 (PEG10) in HCC; however, the correlation between low miR-122 and high PEG10 levels still remains to be defined and require more investigations to evaluate their performance as an effective prognostic biomarker for HCC.

**Methods:**

An in silico approach was used to isolate *PEG10*, a potential miR-122 target implicated in HCC development. miR-122S binding sites in the *PEG10* promoter were evaluated with a reporter assay. The regulation of PEG10 by miR-122S overexpression was examined by quantitative RT-PCR, western blotting, and immunohistochemistry in miR-122 knockout mice and liver tissue from HCC patients. The relationship between PEG10 expression and clinicopathologic features of HCC patients was also evaluated.

**Results:**

miR-122 downregulated the expression of PEG10 protein through binding to 3′-untranslated region (UTR) of the *PEG10* transcript. In miR-122 knockout mice and HCC patients, the deficiency of miR-122 was associated with HCC progression. The expression of PEG10 was increased in 57.3 % of HCC as compared to paired non-cancerous tissue samples. However, significant upregulation was detected in 56.5 % of patients and was correlated with Okuda stage (P = 0.05) and histological grade (P = 0.001).

**Conclusions:**

miR-122 suppresses PEG10 expression via direct binding to the 3′-UTR of the *PEG10* transcript. Therefore, while PEG10 could not be an ideal diagnostic biomarker for HCC but its upregulation in HCC tissue still has predictive value for HCC prognosis.

## Background

Hepatocellular carcinoma (HCC) is the fifth most common cancer around the world and is the cause of nearly 745,000 deaths worldwide each year [1]. Although a number of risk factors have been linked to HCC development, including liver cirrhosis, hepatitis B and C viral infections, and excessive alcohol consumption [2]. HCC is frequently diagnosed at an advanced stage and poor prognosis. The complexity of the disease is due to phenotypic and genetic heterogeneity of affected patients and the multitude of risk factors. Treatment options for HCC are limited and patient resistance to standard chemo- and radiotherapy remains a challenge; in many cases, surgical resection and liver transplantation are the only treatment options. Identifying appropriate biomarkers is critical for early HCC detection and treatment initiation, which can improve survival rates.

Micro (mi)RNAs are expressed in a tissue-specific manner and play essential roles in the regulation of oncogenes and tumor suppressor genes in HCC [3, 4]. miR-122 is specifically expressed in the liver, where it accounts for 70 % of the total miRNA population [4, 5] and regulates lipid metabolism to maintain normal liver function [6, 7]. miR-122 expression was found to be downregulated in HCC tissue, suggesting that miR-122 is associated with hepatocarcinogenesis and can serve as a biomarker for liver cancer [8–15]. miR-122 has many mRNA targets, including *cyclin G1*, *Bcl2*-*like protein 2*, *CCAAT*-*displacement protein*, and *paternally expressed gene 10* (*PEG10*), all of which are overexpressed in HCC patients [12]. These results suggest that miR-122 has a tumor suppressor function in hepatocarcinogenesis. Indeed, mice with targeted deletion of the miR-122 gene exhibited a variety of phenotypes associated with human liver disease, providing a useful model for investigating the effects of miR-122 dysregulation in HCC patients [7].

Aberrant PEG10 expression has been linked to various human malignancies, including pancreatic, breast, and prostate cancers, B cell acute and chronic lymphocytic leukemia [16, 17], as well as HCC [18–21]. PEG10 is critical for parthenogenetic development in mice [22] and is known to stimulate cell proliferation via interaction with activin receptor-like kinase 1 which inhibits transforming growth factor β signaling [23] or with SIAH1, an inducer of apoptosis [20, 24]. Recent studies suggest a role for PEG10 in HCC progression [19, 25–27]; thus, overexpression of PEG10 is considered as a potential biomarker for HCC [28, 29], although the relationship between miR-122 and PEG10 remains not well understood.

In this study, we identified PEG10 as a potential miR-122 target by an in silico approach and suppresses it’s expression via miR-122 direct binding to the 3′-UTR of the *PEG10* transcript. In order to clarify the regulatory interaction between miR-122 and PEG10, the expression levels of these two factors were examined in normal and tumor tissue from HCC patients. Our findings suggest that overexpression of PEG10 can be used to predict HCC patient prognosis at early stages of the disease but may benefit to facilitate therapeutic decision making in HCC.

## Methods

### Plasmid construction

Sense (pSM-miR-122S) and antisense (pSM-miR-122AS) miR-122 expression vectors were provided by Dr. Cliff Ji-Fan Lin [12]. The 3′-untranslated region (UTR) of *PEG10* transcript was cloned into the *Nhe*I/*Xho*I sites downstream of the luciferase gene in the pmiR-GLO plasmid (Promega, Madison, WI, USA) to obtain pmiR-GLO-PEG10-3′-UTR F1 to F5, PEG10 TS (targeted sequence), and PEG10 MTS (mutated targeted sequence). The oligonucleotides contained the restricted linker for the *Nhe*I/*Xho*I-digested terminus and a 47-bp fragment corresponding to the 3′-UTR. The forward 47-bp fragments included a 29-bp upstream flanking sequence, 7- or 8-bp miR-122 seed, and an 11-bp downstream flanking sequence in the 3′-UTR of putative miR-122 targeted genes (Fig. 3c). For mutation analysis, we substituted the 6-nt core seed-matched site (CACTCC) with complementary bases (GTGAGG) (Fig. 3c). The miR-122 positive control targeting sequence, 5′-CTA GCA CAA ACA CCA TTG TCA CAC TCC AGA ATT CAC AAA CAC CAT TGT CAC ACT CCA C-3′, was also cloned into *Nhe*I/*Xho*I sites downstream of the luciferase gene in the pmiR-GLO plasmid to obtain pmiR-GLO-miR122 PTS (positive targeted sequence).

### Cell culture and transfection

Cell culture and DNA transfection was carried out as previously described [30, 31]. HepG2, Hep3B, and 293T cells (HB-8065, HB-8064, and CRL-11268, respectively, from American Type Culture Collection, Manassas, VA, USA) were cultured in Dulbecco’s Modified Eagle’s Medium with 10 % fetal bovine serum and penicillin/streptomycin (Invitrogen, Carlsbad, CA, USA). The 293T cells were transfected using Maxifectin reagent (Omics Biotechnology Co., Taipei, Taiwan). Enhanced green fluorescent protein expression from vectors pSM-miR-122S and pSM-miR-122AS was evaluated with an IX70 fluorescence microscope (Olympus, Tokyo, Japan).

### miR-122 knockout mice

miR-122a^−/−^ mice were provided by Dr. Ann-Ping Tsou (National Yang Ming University). Chimeric mice were produced by crossing with wild-type C57BL/6 mice for germline transmission of the miR-122 allele. Homozygous miR-122^−/−^ mice were obtained by crossing heterozygous offspring [7].

### Patient sample collection

Liver tissue from 147 surgically resected HCC specimens were collected between 1999 and 2015, which stored in the tissue bank of Keelung Chang Gung Memorial Hospital (Keelung, Taiwan). Twelve additional liver tissue samples were obtained by surgical resection of HCC between July 2011 and May 2012 at Keelung Chang Gung Memorial Hospital. HCC cases were selected based on the following criteria: patients over 20 years of age, with or without hepatitis virus infection, who received surgical resection. Exclusion criteria were as follows: the patient or his/her family did not agree to participate in the study; and patients with human immunodeficiency virus infection or other defined etiologies that could lead to liver fibrosis/cirrhosis such as autoimmune hepatitis and alcoholic liver diseases. The research involving human participants in this study was approved by the Multicenter Research and Ethics Committee of Chang Gung Medical Foundation Institutional Review Board. The approval number is 100-0364B. All patients were enrolled after signing and dating an approved informed consent. Patient clinical information was collected according to the approved Institutional Review Board procedures (no: 100-0364B).

### Quantitative real-time PCR

Gene and miRNA expression levels were measured by qRT-PCR as previously described [32]. Total RNA was isolated from cells and tissues with TRIzol reagent (Invitrogen), and 2 μg total RNA was reverse-transcribed into cDNA using SuperScript III (Invitrogen) according to the manufacturer’s instructions. The TaqMan assay (Applied Biosystems, Foster City, CA, USA) was used for qRT-PCR with the 7500 Real-Time PCR System (Applied Biosystems) and default cycling conditions. Relative expression levels were determined from a standard curve generated from serial dilutions of cDNA samples and were normalized to that of *glyceraldehyde 3*-*phosphate dehydrogenase*. Data are presented as histograms where each bar represents the mean ± SEM of data obtained from three independent experiments.

### Reporter assay

The protocol for the reporter assay has been previously described [30]. Briefly, 293T cells were co-transfected with miR-122 (pSM-miR-122S), antisense miR-122 (pSM-miR-122AS), or target reporter plasmid. Luciferase activity was measured with the Dual-Luciferase kit (Promega, Madison, WI) according to the manufacturer’s instructions, and relative protein levels are expressed as firefly/Renilla luciferase ratios.

### Western blotting

Western blotting was carried out as previously described [30–34]. The 293T and HepG2 cells were transfected with the specified concentration of miR-122 (pSM-miR-122S) or anti-sense miR-122 (pSM-miR-122AS), then cultured for 72 h, with PEG10 expression analyzed by western blotting. Total protein was extracted from patient samples and 293T and HepG2 cells using radioimmunoprecipitation buffer with protease and phosphatase inhibitors (Roche Diagnostics, Indianapolis, IN, USA). Lysates were subjected to sodium dodecyl sulfate–polyacrylamide gel electrophoresis and proteins were electrotransferred to a membrane, which was probed with antibodies against PEG10 (4C10A7; Abnova, Taipei, Taiwan) and β-actin (Sigma-Aldrich, St. Louis, MO, USA). Quantity One software (Bio-Rad, Hercules, CA, USA) was used to detect protein bands within the linear range of the scanner, and band intensity was quantified using AlphaImager 2200 software (Thermo Fisher Scientific, Pittsburgh, PA). The ratio of PEG10 to β-actin was used as a quantitative measure of PEG10 regulation by miR-122.

### Histological analysis of PEG10 expression in HCC patient samples

HCC and adjacent normal liver tissue samples were washed twice with phosphate-buffered saline (PBS), then immersed in 4 % paraformaldehyde for 2 h, followed by overnight immersion in 30 % sucrose. Samples were embedded in Optimal Cutting Temperature medium (Electron Microscopy Sciences, Hatfield, PA, USA), frozen on dry ice, and stored at −80 °C. Sections were cut at a thickness of 6–8 μm and placed on glass microscope slides (Thermo Fisher Scientific, Waltham, MA, USA), which were stored at −20 °C. Sections were stained using the Vectastain Elite ABC kit (Vector Laboratories, Burlingame, CA, USA) at room temperature, then permeabilized with 0.5 % Triton X-100, blocked with 1 % bovine serum albumin in 1× PBS and 150 μl stock goat serum for 1 h, and washed with 0.1 M glycine washing buffer. Sections were incubated with polyclonal anti-PEG10 antibody diluted 1:2000 in blocking buffer for 1 h, followed by a 1 h incubation with a biotinylated secondary antibody. The sections were developed for 20 min in a peroxidase substrate solution (Vector Laboratories) and mounted with 50 % glycerol. Phase contrast images were obtained at 60× magnification with an inverted microscope (Olympus).

### Statistical analysis

Continuous variables are expressed as median with interquartile range, while categorical variables are shown as frequencies and percentages. In univariate analysis, baseline characteristics were compared between PEG10 overexpression and control groups with the χ^2^, Fisher’s exact, or Wilcox rank-sum test as appropriate. All reported confidence intervals and tests were two-sided with a 5 % significance level. Data analysis was performed with R v.3.2.4 software (R Foundation for Statistical Computing, Vienna, Austria).

## Results

### miR-122 downregulates PEG10 protein expression

**Fig. 1:**
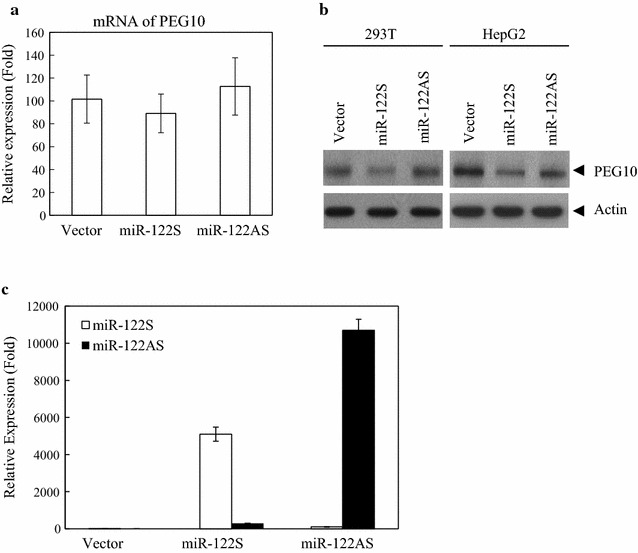
PEG10 is regulated by miR-122 at the post-transcriptional level in 293T and HepG2 cells. **a** PEG10 mRNA levels were detected by qRT-PCR and normalized to that of *glyceraldehyde 3*-*phosphate dehydrogenase* (*GAPDH*) in 293T cells. **b** Western blot analysis of PEG10 expression in 293T and HepG2 cells transfected with miR-122S and miR-122AS. Tubulin was used as the loading control. **c** Overexpression of miR-122 upon HepG2 cells transfection with miR-122S and miR-122AS, as determined by qRT-PCR

To investigate the relationship between miR-122 and PEG10, pre-miR-122 was overexpressed in 293T and HepG2 cells and determined the mRNA and protein expression levels of PEG10 by qRT-PCR and western blotting, respectively. The effect of miR-122 on PEG10 mRNA levels was examined by quantifying the mRNA levels of PEG10 as well as miR-122S and miR-122AS in cells transiently transfected with pSM-miR-122S or pSM-miR-122AS constructs (Fig. 1). The mRNA level of PEG10 was unaltered upon miR-122 or miR-122AS overexpression in 293T cells (Fig. 1a). The protein level of PEG10 was only significantly decreased when miR-122 overexpressed but not miR-122AS in both 293T and HepG2 cells (Fig. 1b, c).

**Fig. 2:**
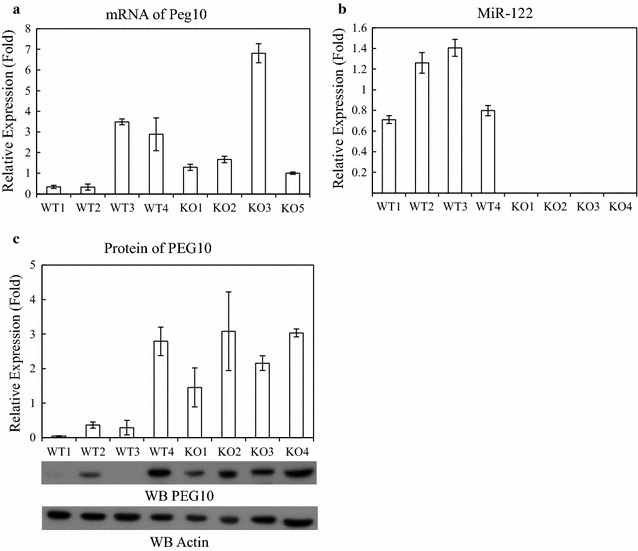
PEG10 is upregulated by miR-122 deficiency in miR-122 knockout (KO) mice. **a** PEG10 mRNA expression levels were determined by qRT-PCR in the liver of wild-type (WT) and miR-122 KO mice and normalized to that of *GAPDH*. **b** miR-122 expression in the liver of WT and miR-122 KO, as determined by qRT-PCR. **c** PEG10 protein level was increased in miR-122 KO as compared to WT mice. Tubulin was used as the loading control

We further to confirm miR-122 mediates the expression of PEG10 protein in vivo, the total RNA and whole cell lysate were extracted from liver tissue of the miR-122 knockout mice and then quantified the relative expression of PEG10 mRNA and protein by qRT-PCR and western blotting, respectively. Figure 2a shows that there is no obvious correlation in mRNA expression level of PEG10 between miR-122-deficient and wild-type mice. In contrast, the protein level of PEG10 was upregulated in miR-122 knockout as compared to wild-type mice (Fig. 2c). These results indicate that miR-122 downregulated the PEG10 expression in liver was at the translational but not transcriptional level.

### Direct interaction of miR-122 with 3′-UTR of PEG10

**Fig. 3:**
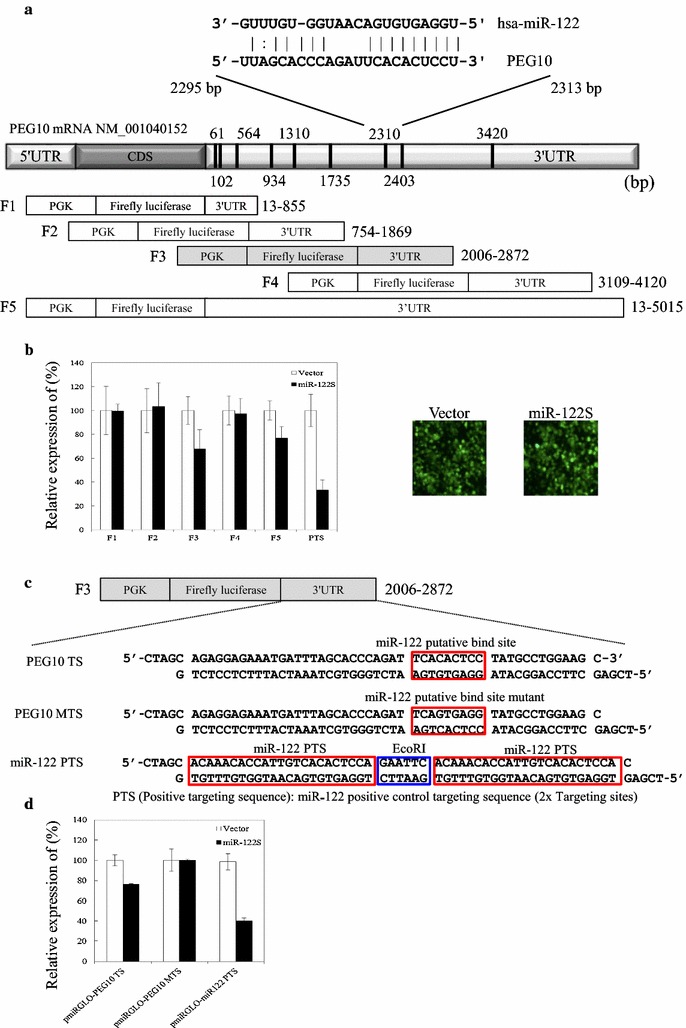
miR-122 directly binds to the 3′-UTR of PEG10 transcript. **a** Schematic illustration of the luciferase reporter vector pmiR-GLO-PEG10-3′-UTR. Nine putative miR-122 binding sites were identified by bioinformatic analysis (*top*) at positions 64, 102, 564, 934, 1310, 1735, 2310, 2403 and 3420. Four deletion fragments and the full-length 3′-UTR of PEG10 were cloned downstream of the luciferase gene at the *Nhe*I and *Xho*I sites. An expanded view of the seed region of miR-122 in the PEG10-3′-UTR is shown. **b** Identification of the miR-122 target region in the 3′-UTR of PEG10 transcript. 293T cells were co-transfected with miR-122S and the negative control (Vector) along with pmiR-GLO-PEG10-3′-UTR F1, pmiR-GLO-PEG10-3′-UTR F2, pmiR-GLO-PEG10-3′-UTR F3, pmiR-GLO-PEG10-3′-UTR F4, pmiR-GLO-PEG10-3′-UTR F5, or pmiR-GLO-miR122 PTS; luciferase activity was determined at 48 h post-transfection. Renilla luciferase served as the internal control. Data represent the mean of three independent experiments, and *error bars* represent SD. **c** Schematic illustration of putative miR-122 binding site in the 3′-UTR of PEG10 transcript. For pmiR-GLO-PEG10 3′-UTR MTS, seven nucleotides, ACACTCC, were replaced with AGTGAGG. *MTS* mutation. **d** Identification of the miR-122 target sequence in the 3′-UTR of PEG10 transcript. 293T cells were co-transfected with either miR-122S or empty vector along with pmiR-GLO-PEG10-3′-UTR TS, pmiR-GLO-PEG10-3′-UTR MTS, or pmiR-GLO-miR122 PTS constructs

In silico analysis using TargetScan and microRNA.org resource (http://www.microrna.org/microrna/home.do). The analysis results indicate that there are nine putative miR-122 binding sites located in the 3′-UTR of PEG10 transcript, i.e., 64, 102, 564, 934, 1310, 1735, 2310, 2403 and 3420 (Fig. 3a). In this study, we performed a luciferase reporter assay to determine whether PEG10 expression is directly mediated by miR-122. The full-length PEG10 3′-UTR and four deletion fragments were subcloned into downstream of the luciferase gene in the pmiR-GLO reporter plasmid (Fig. 3a). Cotransfected 293T cells with each plasmid, along with a miR-122S and measured the luciferase activity at 48 h post-transfection. The data suggest that the miR-122S target sites 2310 and 2403 were found to be responsible for the decreased luciferase activity upon co-transfection with miR-122S (Fig. 3b).

To clarify the interaction between miR-122 and PEG10 3′-UTR, sequences corresponding to miR-122 seed binding sites (Fig. 3c) were constructed [35]. Liver-specific transcription factors regulate miR-122, which in turn targets cut-like homeobox 1 during liver development [35]. The oligonucleotides containing the putative miR-122 binding site(s) were designed and subcloned by ligating annealed oligonucleotides into the *Nhe*I/*Xho*I-digested pmiR-GLO vector in the forward direction (Fig. 3c); the resultant constructs pmiR-GLO-PEG10 TS, pmiR-GLO-PEG10 MTS, and pmiR-GLO 122 PST were transiently transfected into 293T cells. At 48 h post-transfection, the luciferase activity of pmiR-GLO-PEG10 TS was reduced upon co-transfection with miR-122S but not miR-122AS (Fig. 3d). This decrease was abrogated by introducing a 6-bp mutation in the miR-122 binding site. These results indicate that miR-122 downregulates the PEG10 expression via direct binding to site 2310 in the 3′-UTR of PEG10.

### miR-122 is downregulated while PEG10 is upregulated in HCC patients

**Fig. 4:**
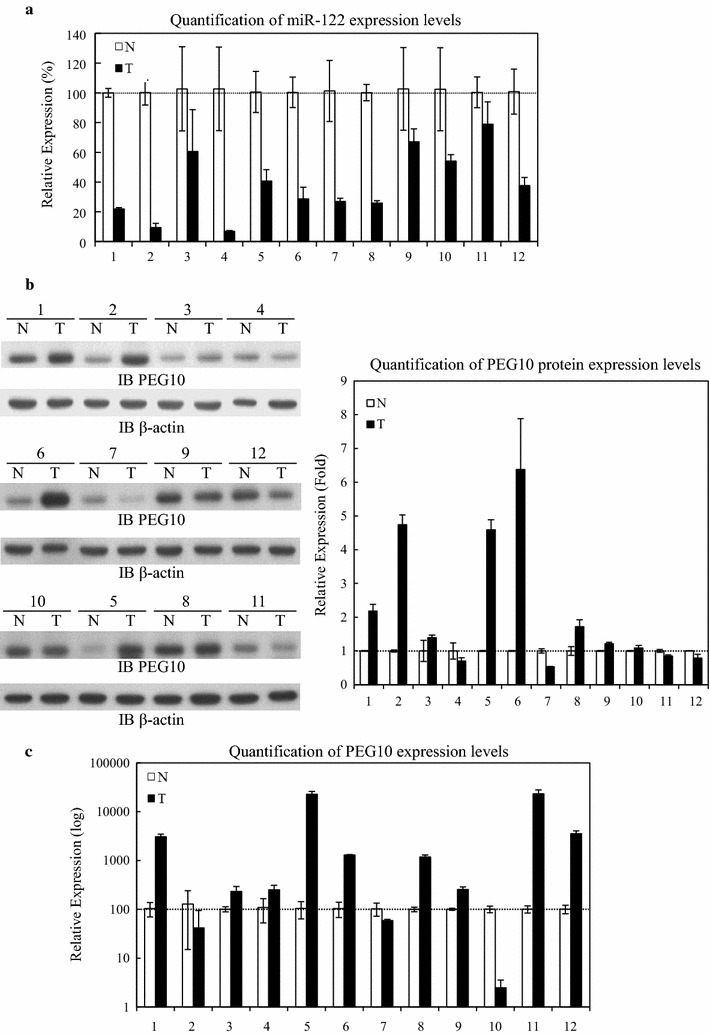
miR-122 and PEG10 expression levels in HCC tissue. **a** miR-122 expression levels in HCC. Total RNA was extracted from 12 paired cancerous and adjacent normal tissues from HCC patients and miR-122 level was quantified by qRT-PCR. **b** Comparison of PEG10 expression in 12 paired cancerous and adjacent normal tissues from HCC patients by western blotting. Data represent the mean of three independent experiments and were normalized to the level of β-actin, and are presented as relative intensity (*right panel*). **c** PEG10 mRNA expression level in cancerous and adjacent normal tissue from HCC patients, as determined by qRT-PCR

miR-122 is downregulated in human HCC and has been considered as part of the miRNA signature for HCC. However, it is still not clear how miR-122 contributes to liver tumor progression. A qRT-PCR analysis of 12 HCC tumors reveal that miR-122 expression levels were downregulated relative to adjacent non-cancerous tissue (Fig. 4a).

PEG10 was previously reported to be associated with increased cell proliferation in HCC patients [20]. In our study, we did not observe significant correlation between the downregulation of miR-122 and relative change of endogenous PEG10 protein levels in HCC (Fig. 4b); only 8 of 12 (67 %) HCC patients had higher PEG10 protein expression in tumor as compared to normal adjacent tissue (Fig. 4b). Accordingly, there was no association between PEG10 mRNA levels in tumor and normal adjacent tissues that examined by qRT-PCR analysis (Fig. 4c). The analysis data revealing that in 9 of 12 (75 %) HCC patients, the PEG10 transcript was overexpressed in tumor tissue. These suggesting that factors other than miR-122 are also involved in regulation of PEG10 expression in HCC.

### PEG10 protein expression is correlated with advanced histological grade and higher Okuda stage and alpha fetoprotein (AFP) level

We analyzed the clinical characteristics of 147 HCC patients from the Tissue Bank of Chang Gung Memorial Hospital in Keelung. Among patients with higher PEG10 protein levels in tumor as compared to normal adjacent tissue, the mean patient age was 62.95 years (range 26–85 years); 58 were male; 40 (48.2 %) were infected with the hepatitis B virus and 28 (33.7 %) with hepatitis C virus; 55 (66.3 %) had cirrhosis of the liver; and 12 (14.5 %) exhibited tumor recurrence. Among patients in which PEG10 protein expression in tumor tissue was similar to or lower than in adjacent normal tissue, mean patient age was 62.31 years (range 37–84); 46 were male; 31 (48.4 %) were infected with hepatitis B virus and 13 (20.3 %) with hepatitis C virus; 42 (65.6 %) had cirrhosis of the liver; and 4 (6.3 %) exhibited tumor recurrence (Table 1).

**Table 1:** Association between PEG10 expression and clinical characteristics in 147 hepatocellular carcinoma patients

	PEG10 (+)	PEG10 (−)	P value
	N = 83	N = 64	
Age (mean)	62.95	62.31	0.662
Gender (M)	58 (69.9 %)	46 (71.9 %)	0.792
Tumor size (cm)			0.231
<2	17 (20.5 %)	7 (10.9 %)	
2–5	39 (47.0 %)	30 (46.9 %)	
>5	27 (32.5 %)	27 (42.2 %)	
Vascular invasion	32 (38.6 %)	25 (39.1 %)	0.950
Liver cirrhosis	55 (66.3 %)	42 (65.6 %)	0.935
AJCC T-stage			0.144
I	49 (59.0 %)	36 (56.3 %)	
II	17 (20.5 %)	11 (17.2 %)	
III	16 (19.3 %)	11 (17.2 %)	
IV	1 (1.2 %)	6 (9.4 %)	
BCLC stage			0.159
0	9 (10.8 %)	2 (3.1 %)	
A	44 (53.0 %)	34 (53.1 %)	
B	26 (31.3 %)	27 (42.2 %)	
C	4 (4.8 %)	1 (1.6 %)	
Okuda stage			0.050
I	79 (95.2 %)	55 (85.9 %)	
II	4 (4.8 %)	9 (14.1 %)	
Etiology			0.173
Non-viral	11(13.3 %)	15 (23.8 %)	
HBV	40 (48.2 %)	31 (48.4 %)	
HCV	28 (33.7 %)	13 (20.3 %)	
Both	4 (4.8 %)	5 (7.8 %)	
Histological grade			0.001
Well	6 (7.2 %)	19 (29.7 %)	
Moderate	61 (73.5 %)	32 (50 %)	
Poor	16 (19.3 %)	13 (20.3 %)	
Recurrence (>2 year)	12 (14.5 %)	4 (6.3 %)	0.113
AFP (>500 ng/ml)	17 (30.1 %)	5 (15.6 %)	0.055

*AJCC* American Joint Committee on Cancer, *BCLC* Barcelona Clinic Liver Cancer, *HBV* hepatitis B virus, *HCV* hepatitis C virus, *AFP* α-fetoprotein

PEG10 protein overexpression was detected in 83 of 147 patients (56.5 %). A univariate analysis revealed that this was significantly associated with advanced histological grade (P = 0.001), higher Okuda stage (P = 0.050), and elevated AFP level (P = 0.055).

## Discussion

miRNAs regulate genes involved in cell proliferation and tissue homeostasis by modulating mRNA stability and protein translation [36]. Accordingly, the dysregulation of miRNAs and their target genes is linked to tumor initiation and progression [37]. The results of our study demonstrated a negative regulatory relationship between miR-122 and PEG10 in two different cell lines. These results are consistent with the study which transient transfected pre-miR122 into HepG2 cells caused a decrease in PEG10 protein level without altering the mRNA level [38]. We also showed with a luciferase reporter assay that miR-122 directly bound to the 3′-UTR of the PEG10 transcript (2006–2872 bp) and further suppress the translation of PEG10.

Previous studies shown that PEG10 highly expressed in hepatoma cell lines and miR-122 downregulated in HCC tissue [4, 7, 39, 40]. These findings imply that expression of miR-122 and PEG10 is inversely related in HCC. We found that miR-122 was expressed at low levels in normal tissue adjacent to tumors in all patient samples, while PEG10 levels varied between specimens. In some cases, PEG10 mRNA was downregulated whereas the protein expression was upregulated relative to normal adjacent tissue, which is consistent with miR-122-mediated translational repression of PEG10; however, there was still no correlation between PEG10 and miR-122 levels in HCC patients. In China and Hong Kong, 39.5 and 80 % of HCC patients, respectively, have increased copy numbers of the *PEG10* gene [26], while PEG10 mRNA is detected in 67, 80 and 67 % of HCC cases in China, Hong Kong, and Taiwan, respectively [19, 26]. In Korea, overexpression of PEG10 protein was detected in 67.9 % of HCC cases, and was correlated with younger age, female, higher Edmondson grade, microvascular invasion, intrahepatic metastasis, higher American Joint Committee on Cancer T stage, and elevated AFP level [25]. By comparison, we observed overexpression of PEG10 protein has been found in 57.3 % of HCC cases in Taiwan. Advanced histological grade, higher Okuda stage, and high AFP level were risk factors for poor survival. The discrepancies between these reported values may be attributed to the existence of more than one mechanism regulating PEG10 expression in HCC.

**Fig. 5:**
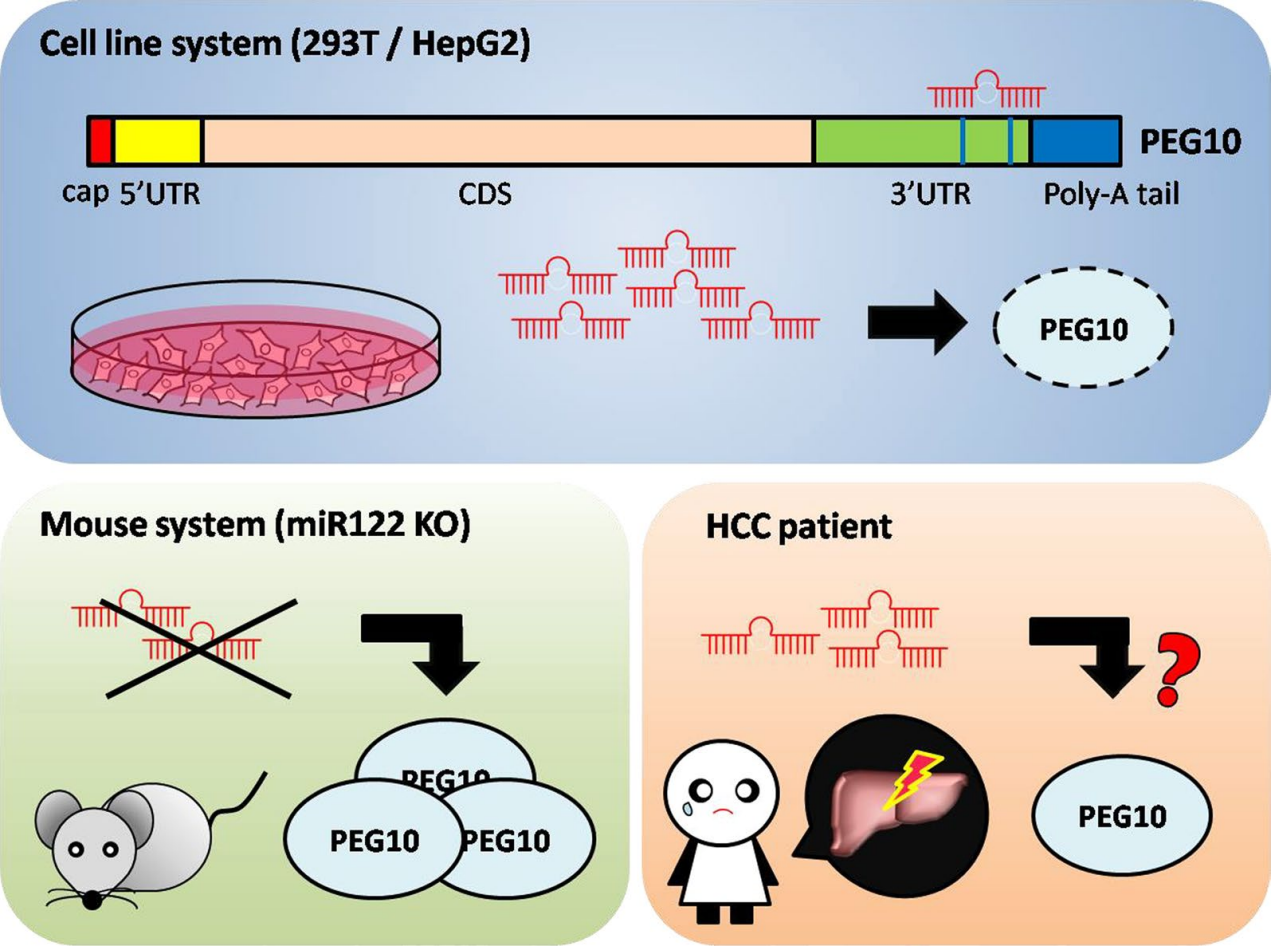
Regulation of PEG10 expression by miR-122 in different model systems. In cell cultures, binding of miR-122 to sites 2310 and 2403 in the 3′-UTR of the PEG10 transcript suppressed PEG10 protein expression. In mice, PEG10 protein level was increased by miR-122 deficiency. In HCC patient tissue, there was no strong relationship between miR-122 and PEG10 levels in normal and tumor tissue, suggesting that other factors regulate PEG10 expression in HCC patients

Our findings demonstrate that miR-122 normally downregulates PEG10 protein expression and this regulation is lost in HCC (Fig. 5), suggesting that the combination of downregulation of miR-122 and upregulation of PEG10 protein can be serve as early biomarkers for identifying an HCC subpopulation that is at high risk for poor outcome.

## Conclusions

miR-122 suppressed PEG10 expression at translation level but not the mRNA level in cell lines and mouse model via direct binding to the 3′-UTR of PEG10 transcript. Significantly, PEG10 protein expression level was positively correlated with advanced histological grade and Okuda stage and high AFP level. Further studies are needed in order to determine whether other factors besides miR-122 regulate PEG10 expression in HCC.

## Abbreviations

AFP: alpha fetoprotein
HCC: hepatocellular carcinoma
miRNA: microRNA
PBS: phosphate-buffered saline
PEG10: paternally expressed gene 10
qRT-PCR: quantitative real-time PCR
UTR: untranslated region
